# Detecting *de novo* Hepatic Ketogenesis Using Hyperpolarized [2-^13^C] Pyruvate

**DOI:** 10.3389/fphys.2022.832403

**Published:** 2022-02-07

**Authors:** Mukundan Ragavan, Marc A. McLeod, Anna Rushin, Matthew E. Merritt

**Affiliations:** Department of Biochemistry and Molecular Biology, College of Medicine, University of Florida, Gainesville, FL, United States

**Keywords:** ketogenesis, hyperpolarization, dichloroacetate, hepatic metabolism, octanoate metabolism

## Abstract

The role of ketones in metabolic health has progressed over the past two decades, moving from what was perceived as a simple byproduct of fatty acid oxidation to a central player in a multiplicity of disease states. Previous work with hyperpolarized (HP) ^13^C has shown that ketone production can be detected when using precursors that labeled acetyl-CoA at the C1 position, often in tissues that are not normally recognized as ketogenic. Here, we assay metabolism of HP [2-^13^C]pyruvate in the perfused mouse liver, a classic metabolic testbed where nutritional conditions can be precisely controlled. Livers perfused with long-chain fatty acids or the medium-chain fatty acid octanoate showed no evidence of ketogenesis in the ^13^C spectrum. In contrast, addition of dichloroacetate, a potent inhibitor of pyruvate dehydrogenase kinase, resulted in significant production of both acetoacetate and 3-hydroxybutyrate from the pyruvate precursor. This result indicates that ketones are readily produced from carbohydrates, but only in the case where pyruvate dehydrogenase activity is upregulated.

## Introduction

Ketones are known to compensate for scarcity of carbohydrates—either from dietary conditions or from a lack of stored glycogen (Werk et al., 1955; Balasse, 1979; McGarry and Foster, 1980). As such, the human liver can produce as much as 300 g of ketone bodies per day for utilization by other organs (Balasse and Féry, 1989). When mammals are subjected to food restriction, peripheral lipids are mobilized into circulation for transport to the liver as substrates for oxidation and, additionally, as substrates for *de novo* ketogenesis. Therefore, hepatic ketogenesis has traditionally been viewed as an adaptive process in response to nutritional deficit (Werk et al., 1955; McGarry and Foster, 1980).

Novel studies of the various effects on energy homeostasis deriving from fatty liver disease have expanded our understanding of ketone body metabolism. For example, recent studies by Go et al. (2016) using a high fat diet (HFD) model of non-alcoholic fatty liver disease (NAFLD) and whole-body pyruvate dehydrogenase kinase 2 (PDK2) knock-out (KO) model demonstrated the interplay between fluxes through pyruvate dehydrogenase (PDH), pyruvate carboxylase (PC), and ketogenesis. This work demonstrated that increased ketogenesis was associated with lower steatosis compared to the HFD model. Similar observation has been reported in humans as well. Fletcher et al. (2019) demonstrated that a decrease in ketogenesis correlated with increasing hepatic steatosis. They further showed that hyperglycemia associated with NAFLD depends not on acetyl-CoA production but rather on its disposal as ketone bodies. These observations suggest that hepatic ketone production is protective and prevents excessive oxidation leading to reactive oxygen species (ROS) generation and consequent mitochondrial dysfunction (Fletcher et al., 2019). While upregulation of PDH flux suggests that a greater proportion of acetyl-CoA derived from carbohydrate sources could have been used as ketogenic substrates, preventing their consumption by citrate lyase and subsequent *de novo* lipogenesis (Li et al., 2015), it still leaves open the question of substrate selection for hepatic ketone production.

From a technical perspective, a method for imaging ketone production might serve as a powerful diagnostic of metabolic dysfunction in the liver. Positron emission tomography (PET) methods are unsuitable for measuring synthesis of new metabolites, as the method itself is insensitive to biochemical transformation and is restricted in large part to measures of uptake. Traditional magnetic resonance imaging (MRI) and spectroscopy (MRS) have the chemical specificity to measure metabolic transformations, but lack the sensitivity to detect ketone production. Fortunately, MR sensitivity can be dramatically enhanced using dissolution dynamic nuclear polarization (dDNP) approaches. DNP can produce “hyperpolarized” (HP) signal enhancements for ^13^C of as much as 40,000 times depending upon the field strength of the detection magnet (Ardenkjær-Larsen et al., 2003). Hyperpolarization has enabled studies of metabolism in cell culture (Harrison et al., 2012; Sriram et al., 2015), perfused organs including heart and liver (Moreno et al., 2010, 2014; Jin et al., 2016; Ragavan et al., 2021), and *in vivo* (Cunningham et al., 2016; Chung et al., 2019).

In this work, we have utilized *ex vivo* mouse liver perfusions, hyperpolarized [2-^13^C] pyruvate, and gas chromatography–mass spectrometry (GC-MS) to characterize hepatic ketone production. We have compared three perfusion conditions—(1) with dichloroacetate, a small molecule PDK inhibitor that has been used to study metabolism in different systems (Hu et al., 2012; Park et al., 2013), (2) with octanoate, a medium-chain fatty acid that does not rely on carnitine palmitoyltransferase-1 (CPT-1) for transport and is avidly oxidized by mitochondria, and (3) mixed fatty acids (FFA), a near-physiological mixture. Treatment with DCA has been shown in previous studies to channel pyruvate utilization toward ketone production, while octanoate is known as a potent ketogenic substrate (McGarry and Foster, 1971) that can have allosteric effects on other oxidative machinery involved in regulation of ketogenesis, such as the branched chain keto acid dehydrogenase (Kadota et al., 2015). While physiological free fatty acids are known to increase ketogenic rates in perfused organ systems in a dose-dependent manner similar to the medium-chain fatty acid octanoate, FFA typically induces less ketogenesis than octanoate (Krebs et al., 1969). Using these groups to modulate hepatic ketogenesis we show that an increase in PDH flux results in a concomitant increase in hepatic ketone production.

## Materials and Methods

### Liver Perfusions

Experiments involving C57/BL6N mice were handled in compliance with University of Florida Institutional Animal Care and Use Committee approved protocol (#201909320). Liver perfusions were carried out as described in the literature (Moreno et al., 2014; Ragavan et al., 2021). Briefly, male mice (10–13 weeks old) in the fed state were anesthetized using isoflurane followed by an intraperitoneal injection of heparin. Approximately 10 min after heparin injection, lidocaine was administered subcutaneously followed by a celiotomy under anesthesia to expose the liver and portal vein. The portal vein was cannulated, and perfusion was started. The liver is then excised from the body and connected to a glass perfusion column. This glass perfusion apparatus is then moved into the bore of the NMR magnet.

### Experimental Groups

Liver metabolism was studied in three groups—(1) *n* = 5, free fatty acid (FFA), (2) *n* = 4, octanoate, and (3) *n* = 5, octanoate plus dichloroacetate (DCA). Perfusate used in all groups contained Krebs–Henseleit electrolytes (25 mM NaHCO_3_, 112 mM NaCl, 4.7 mM KCl, 1.2 mM each of MgSO_4_ and KH_2_PO_4_, 0.5 mM sodium-EDTA, and 1.25 mM CaCl_2_), 1 mM sodium lactate, and 0.1 mM sodium pyruvate. FFA group contained 0.63 mM mixed fatty acids [containing palmitic acid (22.1% of total), palmitoleic acid (5.2%), stearic acid (2.7%), oleic acid (27%), linoleic acid (37.7%), γ-linolenic acid (2.4%), and docosahexaenoic acid (2.8%)] along with 2% (w/v) bovine serum albumin. The octanoate group contained 0.1 mM sodium octanoate while octanoate plus DCA group contained 0.1 mM octanoate with 20 mg/ml dichloroacetate.

Perfusate was oxygenated using 95% O_2_/5% CO_2_ mixed gas for the duration of perfusion. Hepatic oxygen consumption during perfusion was measured periodically (approximately every 15 min) using an Oxygraph+ setup (Hansatech Instruments, United Kingdom). At the end of perfusion, the livers were freeze-clamped in liquid nitrogen and stored at −80°C until needed.

### Dynamic Nuclear Polarization

[2-^13^C] pyruvic acid (Cambridge Isotope Laboratories, United States) was doped with 15 mM trityl radical (OX063; tris[8-carboxyl-2,2,6,6-tetra-[2-(1-hydroxyethyl)]-benzo-(1,2-d:4,5-d)-bis-(1,3)-dithiole-4-yl]-methyl sodium salt; Oxford Instruments, United Kingdom) and 1 mM ProHance. This sample was inserted into a HyperSense polarizer (Oxford Instruments, United Kingdom). The frozen sample was hyperpolarized at 1.4 K by applying microwave irradiation (94.116 GHz at 100 mW) until steady state polarization was achieved (~1–1.5 h).

The sample was dissolved using 4 ml of hot 20 mM PBS (pH 7.4) and rapidly transferred to a 14 T NMR magnet. 3 ml of the dissolved sample was mixed with 20 ml of Krebs–Henseleit electrolytes and injected directly into the perfused liver (nominal final concentration of HP pyruvate is 4 mM). Injection of HP substrate as a bolus was achieved with the use of long tubing running along the length of the glass perfusion column and terminating immediately above the catheter. Substrate enters the liver *via* the catheter placed in the portal vein. NMR signal acquisition was started prior to injection.

### NMR Spectroscopy

Spectra in HP pyruvate experiments were collected in a custom-built 20 mm broadband probe (QOneTec, Switzerland) installed in a 14 T magnet equipped with an Avance III NMR console (Bruker Biospin, United States). Shimming was carried out using the ^23^Na signal prior to the HP experiment. Linewidths of ~18 Hz in ^23^Na spectrum were achieved. ^13^C spectra (spectral width of 300 ppm; 32,768 data points) were recorded using 30° radiofrequency pulses with ^1^H decoupling (WALTZ65) during acquisition and a repetition time of 3 s. Spectra were processed with 20 Hz exponential line broadening and baseline corrected using a polynomial function. Peak areas of metabolites were obtained by fitting mixed Lorentzian-Gaussian line shapes. NMR spectra were processed using MestReNova (v14.2.1).

### Gas Chromatography–Mass Spectrometry

#### Perfusate Extraction

Protein in 1 ml of perfusate was precipitated by adding 100 μl of 70% (v/v) perchloric acid. Samples were centrifuged. 1 ml of supernatant was collected and pH adjusted to 7 using 5 M KOH. The samples were centrifuged to pellet out potassium perchlorate salt before collecting 800 μl of supernatant. The supernatant was dried in a lyophilizer (Thermo Scientific Waltham, MA). The dried solution was reconstituted in 400 μl of 50:50 Acetonitrile water and then allowed to sit in a −20°C freezer for at least 2 h before centrifugation and 330 μl of the resulting supernatant was collected. All centrifugation steps were carried out at 4°C and 10,000 × *g* for 30 min.

#### Liver Extraction

Freeze-clamped perfused liver samples were stored at −80°C prior to analysis. 35 +/− 3 mg of perfused livers were transferred into conical bottom microcentrifuge tubes with screw caps. 1.0 mm zirconium oxide homogenization beads were added to each microcentrifuge tube along with 400 μl of cold degassed acetonitrile:isopropanol:water (3:3:2 v/v/v) solvent mixture. Samples were homogenized with a bead homogenizer (Fastprep-24, M.P. Biomedicals, Irvine, CA) for 3 × 20 s cycles and were cooled on ice for 5 min between each cycle. The samples were then centrifuged at 10,000 × *g* at 4°C for 30 min. 300 μl of supernatant was recovered and lyophilized (Thermo Fisher Scientific, Waltham, MA, United States). The dried precipitate was reconstituted in 100 μl of acetonitrile:water (1:1 v/v) mixture, followed by incubation at −20°C for at least 2 h, centrifugation at 10,000 × *g* at 4°C for 30 min and recovery of 80 μl of supernatant. Reagents, unless otherwise specified, were purchased from Fisher Scientific, Waltham, MA, United States.

#### MTBSTFA Derivatization

For analysis of ketones, glycolytic end products, amino acids, and TCA cycle intermediates, ~8 mg of liver extract (30 μl) or 100 μl of perfusate or 50 μl of FFA perfusate extract was dried down by airstream. The resulting powder was reconstituted in 50 ul of methoxyamine HCL in pyridine (Thermo Fisher Scientific, Waltham, MA, United States) for 1.5 h at 30°C, while stirred by microstirbar. Then 50 μl of dimethyl tert butyl silyl trifluoroacetamide (MTBSTFA; ProteoSpec MTBSTFA w/1% TBDCMS, Ricca Chemical Company, Arlington, TX, United States) was added and allowed to react at 70°C for 30 min while stirring.

#### GC-MS Method

After MTBSTFA derivatization was complete, 5 μl of each liver sample or perfusate sample was pooled together to make a “pooled sample” and 80 μl of each individual sample was loaded into a 150 μl glass insert (CAS#13-622-207, Thermo Fisher scientific, Waltham, MA, United States) inside of a GC vial with a 9 mm PTFE/red rubber septum (CAS#C4000-30, Thermo Fisher Scientific, Waltham, MA, United States). The samples were loaded onto the AI/AS 1300 autosampler to await auto injection into the Trace1310 Gas chromatograph system (Thermo Fisher Scientific, Waltham, MA, United States). Samples of 1 μl volume were injected into the injection port with a splitless single taper liner at 250°C and a splitless time of 60 s and column flow at 1 ml/min with a 30 m RTX-5MS integra Guard column (Crossbond 5% diphenyl/95% dimethylpolysiloxane CAT# 12623-127, Restek, Bellefonte, PA, United States) and 10 m guard column. The injected samples were held at 60°C for 1 min and were followed by a ramp of 15°C/min up to 320°C followed by a 5 min bakeout at 320°C. For all samples, the transfer line was maintained at 280°C and the ion source was maintained at 230°C. The solvent delay on the mass spectrometer was set to 7.5 min for all samples. A *m*/*z* filter of 40–600 mass-to-charge ratio was used, and individual metabolites and their respective isotopologue distributions were quantified, by area, using their known quantification ions as in Supplementary Table S1.

##### Quantification

Peak areas were integrated using Xcalibur (version 4.1) batch processing with genesis peak fitting, a 5-s time interval, height cutoff at 5.5% of the peak with valley detection enabled, and a S/N cutoff threshold of 3. Concentrations for metabolites were determined using a 6-point standard curve with a range of 200–2,000 ng/sample or 200–3,500 ng/sample per metabolite for liver and perfusate, respectively. Fractional enrichments were calculated using isotopomer network compartment analysis (INCA) and the predicted natural abundance distribution by chemical formula of the mass fragments analyzed (Fernandez et al., 1996; Young, 2014).

### Statistical Analysis

Statistical significance was established using one-way ANOVA with post-hoc analysis using Tukey HSD. *p*-values of less than 0.05 were considered significant.

## Results

**Figure 1:**
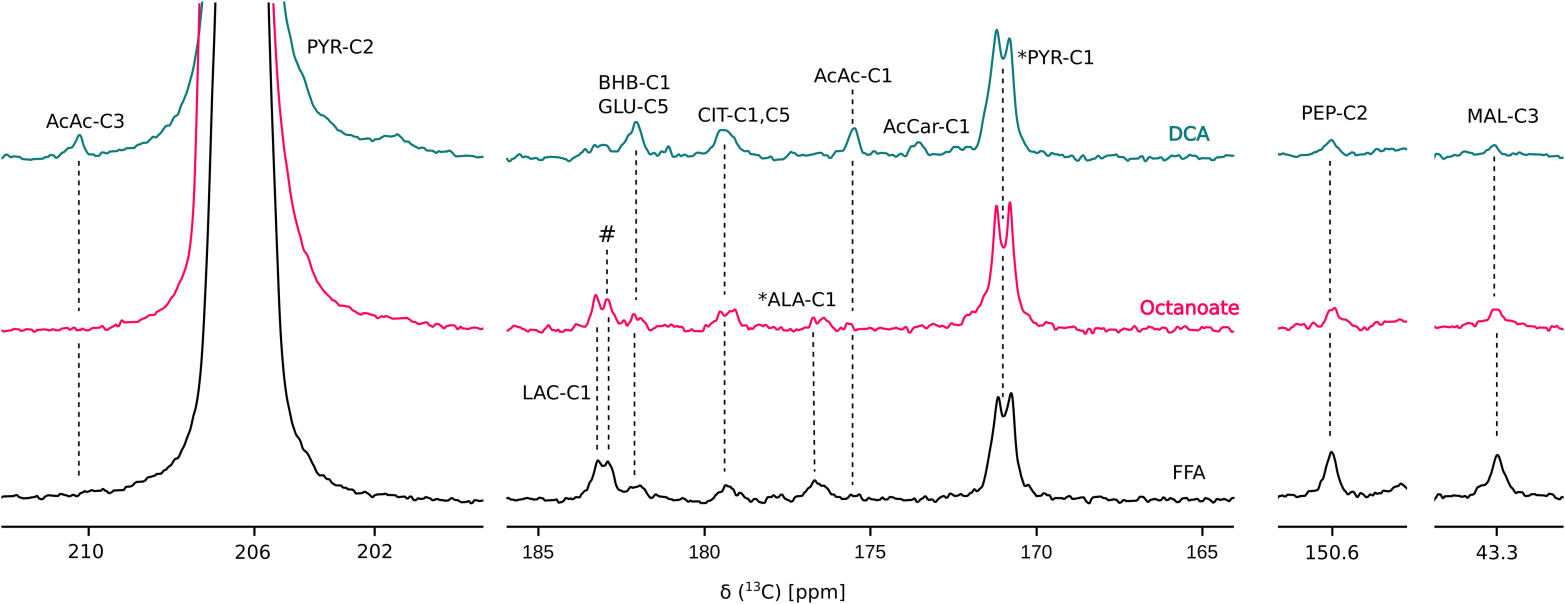
Overlay of representative ^13^C sum spectra following injection of HP [2-^13^C] pyruvate. AcAc, acetoacetate; AcCar, acetyl carnitine; ALA, alanine; BHB, 3-hydroxy butyrate; CIT, citrate; GLU, glutamate; LAC, lactate; and PYR, pyruvate. ^#^ and * represent unidentified and natural abundance signals, respectively. “DCA” is Dichloroacetate. Perfusion conditions and NMR parameters are described under Materials and Methods section. Resonance assignments were confirmed using [^1^H, ^13^C] HSQC and [^1^H, ^13^C] HMBC experiments.

**Figure 2:**
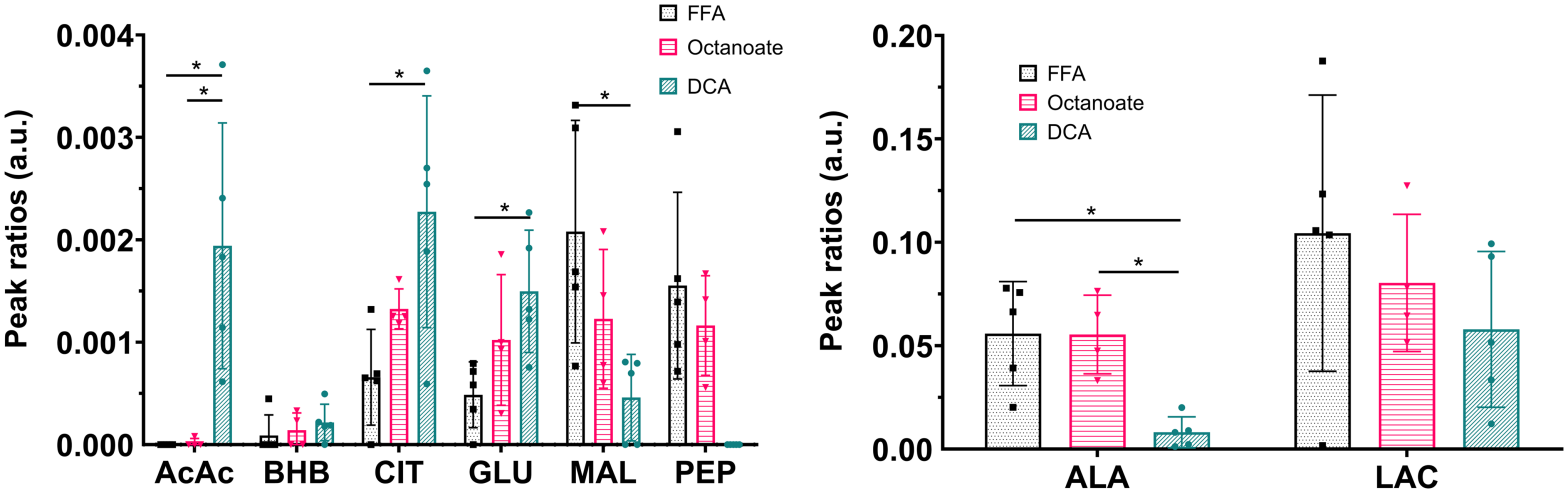
Ratios of signal intensities of individual resonances to sum of all ^13^C resonances in the spectrum. Pyruvate-hydrate resonance was excluded from total ^13^C intensities sum. *Indicates statistical significance (*p* < 0.05; see section Materials and Methods). AcAc sum includes signal intensities at both C-1 and C-3 chemical shifts. AcAc, acetoacetate; AcCar, acetyl carnitine; ALA, alanine; BHB, 3-hydroxy butyrate; CIT, citrate; GLU, glutamate; MAL, malate; LAC, lactate; PEP, phosphoenolpyruvate; and PYR, pyruvate. *n* = 5 (DCA), 4 (Octanoate), and 5 (FFA).

Liver mass and oxygen consumption values were similar across all three groups (Supplementary Figure S1). Hyperpolarized [2-^13^C] pyruvate is readily utilized and converted to various downstream metabolites including acetoacetate (AcAc), glutamate, 3-hydroxybutyrate (BHB), citrate, phosphoenolpyruvate (PEP), malate, alanine, and lactate (Figures 1, 2). In the DCA group, significantly higher amounts of AcAc were observed compared to octanoate and FFA groups. Signals corresponding to ^13^C label in both C-1 and C-3 positions of AcAc were observed in spectra for DCA group. While citrate and glutamate peak intensities were significantly higher in the DCA group compared to FFA livers, malate produced from pyruvate was lower (Figure 2). While AcAc signal was elevated, the redox coupled BHB signal trended higher, but was not significantly different between groups. Malate and PEP signals can only be produced *via* flux through pyruvate carboxylase. While the liver preferentially carboxylates pyruvate (Merritt et al., 2011) PDH activation reduces the intensity of the signals associated with malate and PEP.

**Figure 3:**
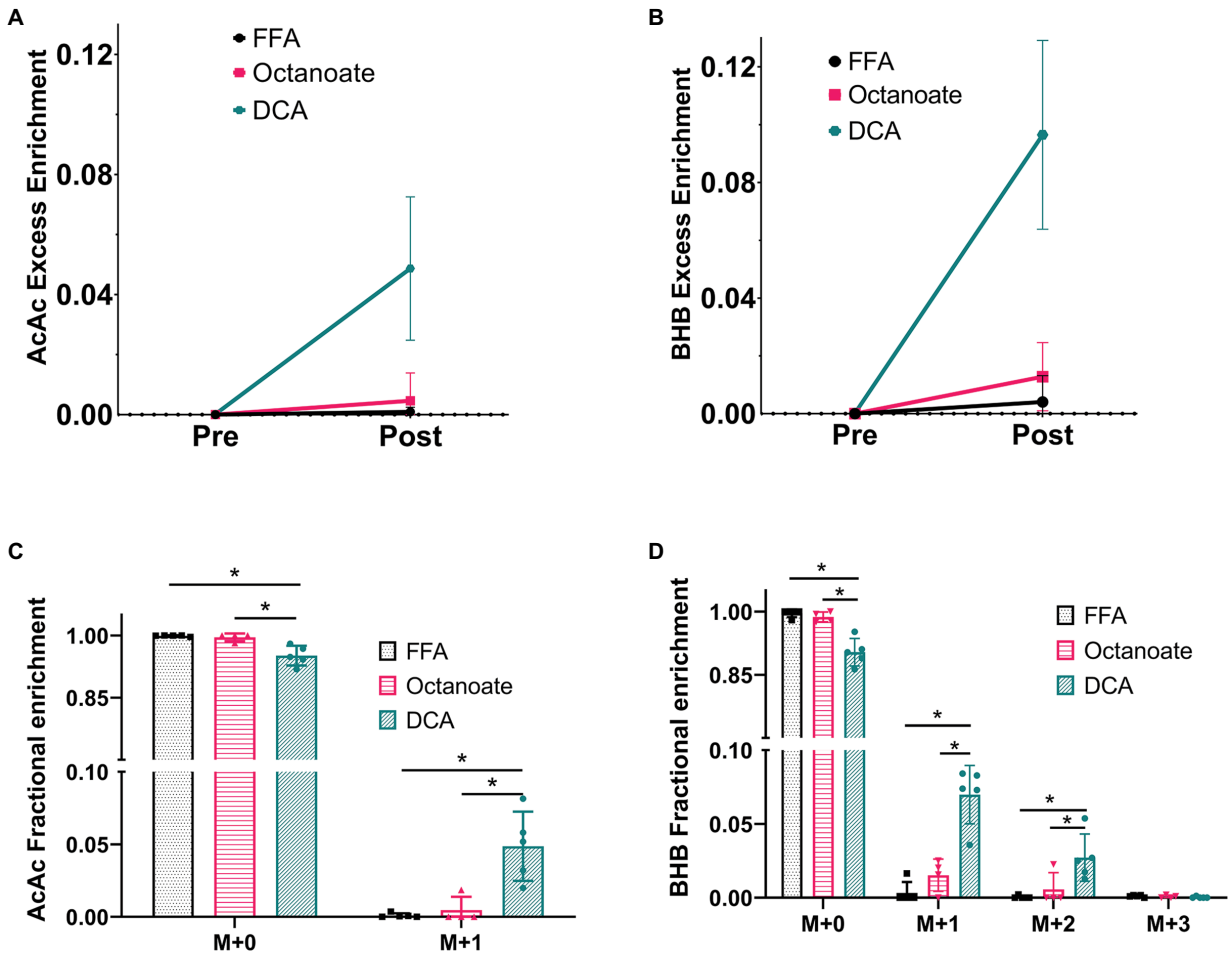
Fractional enrichments for the ketones Acetoacetate and 3-hydroxybutyrate based on GC-MS analysis of efferent perfusate. The data shown is presented after natural abundance correction using the INCA software, which results in no excess enrichment prior to the injection. **(A,B)** Enrichments for ketones produced prior to [2-^13^C] pyruvate were confirmed to contain no label while ketone enrichment was observed to increase in the octanoate and DCA groups considerably indicating labeled ketone production from [2-^13^C] pyruvate. **(C,D)** The fractional enrichment profile for Acetoacetate (M + 0–1) and BHB (M + 0–3) showed significantly higher label in the DCA group than the FFA and octanoate groups for all but BHB M + 3. BHB M + 3 cannot be produced from [2-^13^C] pyruvate and hence has an enrichment within the error of measurement ~0.1% or 0.001. *Indicates statistical significance (*p* < 0.05; see section Materials and Methods).

Hyperpolarized ^13^C spectra, while sensitive to ketones produced from [2-^13^C]pyruvate, do not report on the total enrichment of ketones achieved by metabolism of the labeled substrate. To estimate enrichment of ketones produced by the livers, GC-MS analysis of perfusate collected prior to and post HP [2-^13^C]pyruvate administration was performed (Figure 3). In the DCA group, ^13^C enrichments of more than 5% were achieved for both AcAc and BHB (Figures 3A,B) post HP [2-^13^C]pyruvate injection. Less than 1 and 3% ^13^C enrichments were obtained for ketones in the FFA and octanoate groups. The AcAc major product peak overlaps with DCA in the gas chromatogram, however, the minor peak has no such overlap and can be used for fractional enrichment determination. Since we were forced to use the minor product peak, the M + 2 and M + 3 isotopologues of the AcAc minor peak could not be detected reliably using GC-MS due to low intensity (Figure 3C; Supplementary Figure S2).

**Figure 4:**
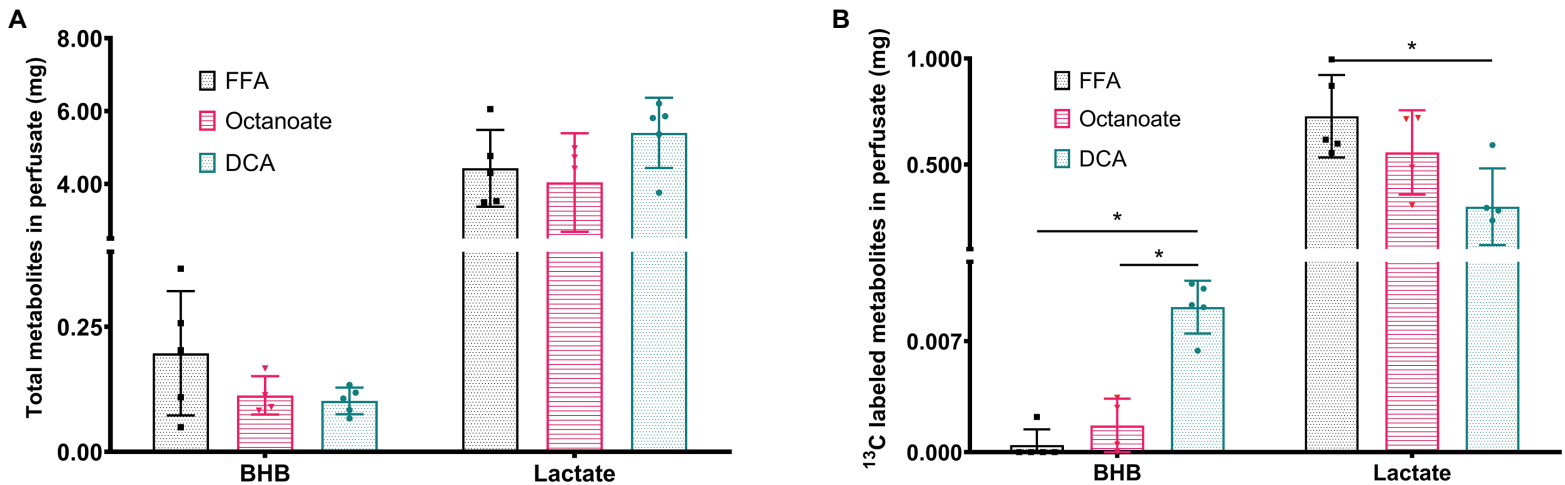
**(A)** Total amount of BHB and Lactate from efferent perfusate post [2-^13^C] pyruvate injection in mg using GC-MS standard curve quantification. **(B)** Amount of ^13^C labeled BHB and Lactate that are (total mg metabolites * total enrichment) in efferent perfusate post [2-^13^C] pyruvate injection. *Indicates statistical significance (*p* < 0.05; see section Materials and Methods).

**Figure 5:**
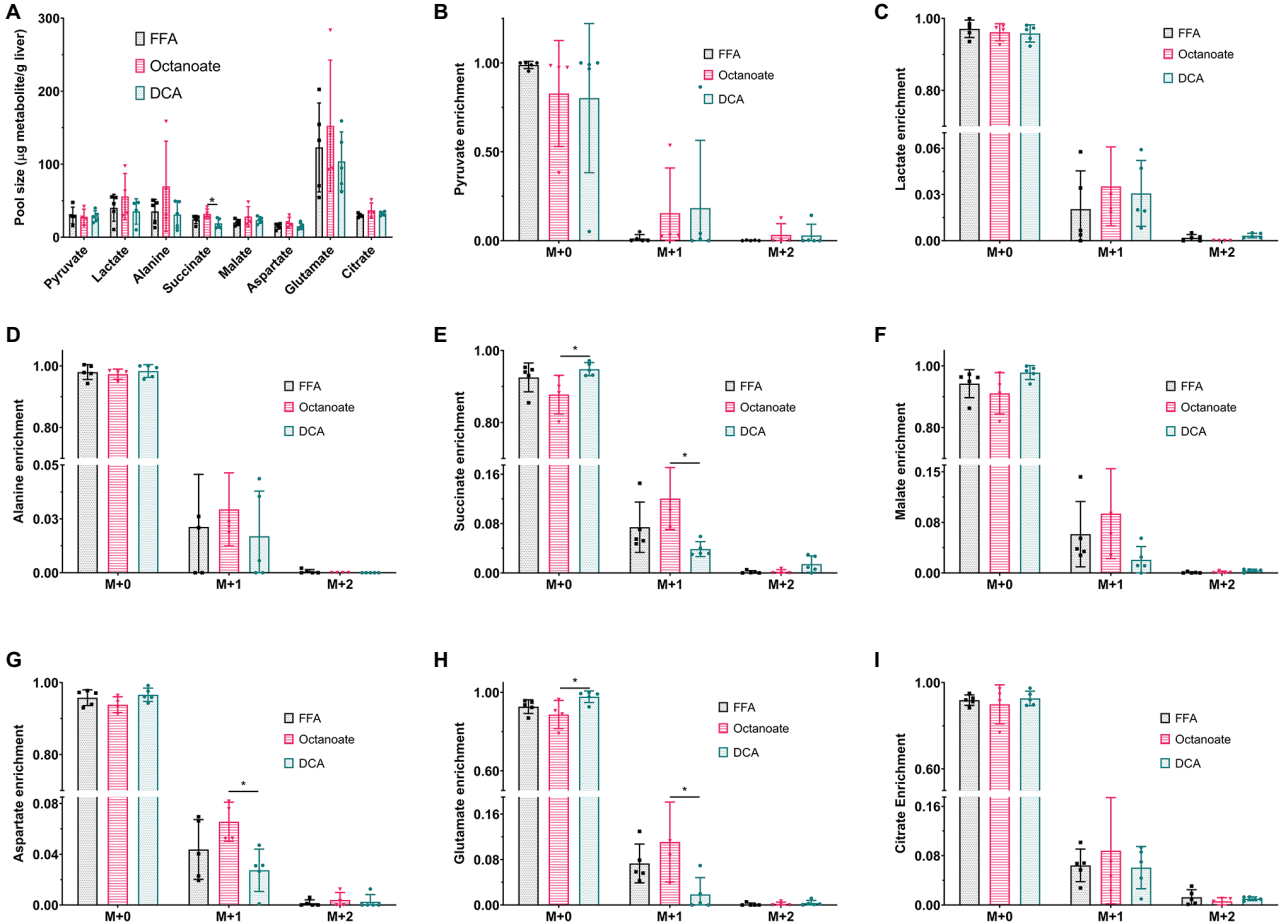
**(A)** Pool sizes of metabolites extracted from the liver estimated using GC-MS. **(B**–**I)** ^13^C enrichment of all metabolites shown in **(A)**. M + 0, M + 1, M + 2, and M + 3 represent individual mass isotopologues. *Indicates statistical significance (*p* < 0.05; see section Materials and Methods).

Although total BHB production as measured from the liver effluent was not significantly different between groups, ^13^C labeled BHB in DCA treated livers was significantly higher than both the FFA and octanoate groups (Figures 3D, 4). Conversely, the ^13^C labeled level of lactate in the DCA group was significantly lower than the FFA group (Figure 4) without an apparent change in perfusate lactate pool size (Figure 5A). The increased ^13^C labeled BHB production from pyruvate-derived acetyl-CoA did not result in a concomitant increase in pool size for TCA cycle intermediates in extracted livers (Figures 5A–I) between different groups, but did show a significant decrease in the succinate pool size between octanoate and DCA groups. Enrichments in the TCA cycle were found to be relatively constant between FFA and DCA groups but the DCA enrichments for succinate, glutamate and aspartate were significantly lower than that of the octanoate groups. Given the similarities in O_2_ consumption between all groups (Supplementary Figure S1), large changes in TCA cycle turnover are not caused by the interventions.

## Discussion

Hepatic ketone production serves to provide a readily oxidizable substrate for multi-organ energy homeostasis (Puchalska and Crawford, 2021). Canonically, β-oxidation has been assumed to provide the vast majority of acetyl-CoA used for ketone production (Puchalska and Crawford, 2017). Whereas here, it is shown that carbons associated with carbohydrate metabolism can effectively trace *de novo* ketogenesis as well. Pyruvate transport into the cells is handled by the high-throughput MCT-1 transporter, which can dominate the appearance of lactate in cancer, but appears to have minimal control over the appearance of metabolites in the liver (Rao et al., 2020). Multiple fates of pyruvate include oxidative metabolism and/or conversion to alanine *via* alanine transaminase (ALT-2) reaction in the mitochondria, or to alanine (ALT-1) or lactate *via* lactate dehydrogenase in the cytosol. DCA has been used in multiple instances in the HP pyruvate literature, in both cases producing downstream HP AcAc signals from tissues that are not normally recognized as highly ketogenic (Park et al., 2013, 2021). In both previous cases, HP AcAc was labeled at both the C1 and C3 positions, indicative of *de novo* ketogenesis versus pseudo-ketogenesis, which is known to operate in tissues outside the liver (Fink et al., 1988). Pseudo-ketogenesis would be characterized by production of the [1-^13^C]-labeled ketones only (Bastiaansen et al., 2016).

In this study, it is evident that the presence of DCA in the perfusate promotes pyruvate dehydrogenase flux as well as increased deposition of HP ^13^C into the ketones and products of oxidative metabolism (Figure 2). In addition to ketone production from HP pyruvate, citrate, glutamate, and malate signals were also significantly different between DCA and FFA groups. While ^13^C label observed from HP spectra in citrate and glutamate arises from oxidative flux, ^13^C label in malate arises due to pyruvate carboxylase flux. Lower ^13^C signal intensities of malate in the HP spectra suggest lower PC flux in the presence of DCA, as expected due to increased production of acetyl-CoA from pyruvate. Similarly, ^13^C alanine produced from HP [2-^13^C] pyruvate is significantly lower in DCA group compared to both octanoate and FFA groups suggesting reduced flux though alanine transaminase under ketogenic conditions. GC-MS results indicate more pyruvate is funneled into ketogenesis as opposed to the TCA cycle (Figures 3, 5), at the expense of lactate production (Figure 4). Despite the known ketogenic properties of octanoate, the HP AcAc and BHB signals were not significantly larger than controls in the octanoate perfusions. This is likely indicative of the competition between octanoate and pyruvate for production of acetyl-CoA, as the GC-MS did not show increased labeling of the ketones either.

HP spectra report on production of ^13^C labeled metabolites as pyruvate is being introduced to the liver, weighted by T_1_ relaxation of the hyperpolarized state. GC-MS, on the contrary, provides an average measurement of the metabolic state of the liver at the end of perfusion—about 5 min after addition of pyruvate. Due to the different times of sampling, ^13^C enrichment reported from GC-MS represents multi-pass labeling (i.e., several turns of citric acid cycle are possible) established in the liver. Furthermore, ^13^C spectra provide information on positional enrichment of metabolites whereas GC-MS data does not provide the same information. These differences are reflected in the apparent contradiction between NMR-derived information on metabolites like citrate and malate compared to GC-MS derived values. HP spectra suggest elevated ^13^C incorporation in citrate and glutamate in DCA group compared to FFA group (Figure 2) while there is no difference in ^13^C enrichment according to GC-MS data (Figure 5). These would likely be different if the liver was perfused to isotopic steady state. There has been some criticism of HP approaches for measuring metabolic flux, as the bolus of material is assumed to be perturbing to the metabolism. On the contrary, these results are consistent with HP essentially holding a unique ability to assess metabolism. While a bolus of pyruvate ultimately can influence overall metabolism, the short time frame for reading the hyperpolarized experiment essentially causes the experiment to function as a tracer experiment. If the carbon-13 T_1_s were *longer*, it would make the experiment more difficult to interpret due to changes in metabolic pool sizes caused by the bolus. Another aspect of HP studies is the effect of spin lattice relaxation times on the observed intensities. In this study, most of the resonances considered are carboxyl or ketone moieties that can be reasonably expected to have similar T_1_ values. Although C-2 of lactate and alanine will have shorter T_1_ compared to other resonances, they will be similar to each other. Further, our use of GC-MS enables accurate quantification of pool size and enrichment without the need to account for differences in T_1_.

The serum or perfusate ratio of BHB/AcAc has been typically measured between ~0.6–3 in fasting and non-fasting animals with fasting increasing the ratio of BHB/AcAc even up to 7 (Krebs et al., 1969; Deja et al., 2021). It is canonically appreciated that the increase in the BHB/AcAc ratio during fasting relates to the change in redox state from excessive oxidative metabolism and has important connotation in diseases, such as NAFLD (Fletcher et al., 2019). The use of hyperpolarized NMR in this study was sensitive to an increase in *de novo* ketogenesis during DCA treatment that is primarily driven by carbohydrate oxidation (Figures 2–4). However, we were not able to assess whether the ratio of BHB/AcAc increased due to low AcAc minor peak intensities and failed attempts to consistently quantify the minor mass peak accurately using external standards. This issue arises primarily because of the relative instability of AcAc in solution. This is unfortunate, as the HP data suggest that AcAc production *via* the acetoacetyl-CoA thiolase reaction is significantly faster than the redox-dependent activity of β-hydroxybutyrate dehydrogenase. DCA treatment results in production of both C1 and C3 labeled AcAc signals that are much greater than the corresponding BHB peak monitored at ~182 ppm, which is deconvoluted from the [5-^13^C]glutamate signal. Even normalizing by a factor of 2 to account for the number of carbons contributing to the resonances would result in excess AcAc signal versus the BHB. Because of the time resolution enabled by HP MR, we can directly observe the relative activities of the thiolase and BHB dehydrogenase enzymes. It is remarkable that DCA display such an incredible propensity for the induction of *de novo* ketogenesis. Our data, along with that previously seen in the brain and muscle from other researchers, indicate that ketogenesis from carbohydrates is significantly upregulated by activation of PDH. Future experiments stimulating PDH activity might test if the induction of ketogenesis would have a protective effect in fatty liver disease (Fletcher et al., 2019). In that case, these observations could serve to establish a baseline effective dosage of the therapeutic agent.

### Limitations

The primary limitation of this study is the *ex vivo* perfusions utilized. The experimental setup lacks the hormonal control that is always present *in vivo*. Additionally, no glycerol was used in the perfusate to avoid substrate competition with hyperpolarized pyruvate. Due to the nature of the HP experiments, absolute metabolic turnover cannot be estimated in the present studies. The overall intensity of the ketone resonances was much lower than expected based on previous *in vivo* results in the brain and heart (Park et al., 2013, 2021; Bastiaansen et al., 2016). With the well-known propensity of octanoate for the induction of ketogenesis (McGarry and Foster, 1971), we assumed the hormonal milieu would not be a controlling factor in our observations. Glucagon is a known agonist of ketogenesis (Bewsher and Ashmore, 1966; Williamson et al., 1966), but it was not included in the perfusates. As previous results showed very large ketone signal in tissues that are not readily acknowledged as having significant ketogenic capacity, we assume that our study reproduced *in vivo* in the liver would result in significantly higher signals arising from AcAc and BHB. While hepatic production of ketones from HP pyruvate is still to be shown in future experiments, we believe that this approach should be sufficient to observe ketone production in humans.

Acetoacetate is a labile molecule with a known propensity to breakdown to acetone and CO_2_ under STP conditions. Despite this, an appreciable quantity of acetoacetate remains in perfusate samples that can be chemically modified through derivatization into a more stable form which appears as a major and minor peak in GC-MS analysis. Unfortunately, derivatized DCA and AcAc major peak have the same retention time and similar mass spectrum hindering quantification. This requires the use of the less intense minor peak for accurate quantification and has prevented analysis of acetoacetate concentration and enrichments above the M + 1 isotopologue due to low intensities specifically in the DCA group but not the FFA or octanoate groups. With adjustments to the type of chemical modification such an overlap between acetoacetate and DCA could be prevented in the future.

## Conclusion

To conclude, we have demonstrated a robust method of detecting ketogenesis using hyperpolarized [2-^13^C] pyruvate and *ex vivo* liver perfusions. The protective benefit of ketogenesis for liver health is posited (Cotter et al., 2014). If downregulation of hepatic ketogenesis is truly a hallmark of NAFLD progression, a means of measuring ketogenesis *in vivo* might serve as a diagnostic of the NAFLD to non-alcoholic steatohepatitis (NASH) transition. This transition causes a change in medical treatment on diagnosis. However, the most effective means for determining this transition, liver biopsy, is prone to sampling error and has a non-trivial risk of mortality. A metabolic imaging method would allow organ specific assessment of changes associated with the NAFLD-NASH transition and could be easily combined with other imaging methods to produce a multi-parametric assessment. Furthermore, an imaging method would provide information on metabolic zonation and logically would also be sensitive to the fibrotic state of the tissue.

## Data Availability Statement

The raw data supporting the conclusions of this article will be made available by the authors, without undue reservation.

## Ethics Statement

The animal study was reviewed and approved by the University of Florida Animal Care and Use Committee.

## Author Contributions

MR and MEM designed the study. MR, MAM, and AR performed liver perfusions. MR performed NMR spectroscopy. MAM performed GC-MS. MR and MAM analyzed the data and wrote the initial draft. MEM acquired funding, supervised the study, and edited the manuscript. All authors contributed to the article and approved the submitted version.

## Funding

This work was supported by funding from National Institutes of Health (R01-DK105346, P41-GM122698, and 5U2C-DK119889). AR was supported by NIH T-32 DK10876.

## Conflict of Interest

The authors declare that the research was conducted in the absence of any commercial or financial relationships that could be construed as a potential conflict of interest.

## Publisher’s Note

All claims expressed in this article are solely those of the authors and do not necessarily represent those of their affiliated organizations, or those of the publisher, the editors and the reviewers. Any product that may be evaluated in this article, or claim that may be made by its manufacturer, is not guaranteed or endorsed by the publisher.
