# Supplementary material for: Detecting de novo Hepatic Ketogenesis Using Hyperpolarized [2-13C] Pyruvate
Source: Front Physiol. 2022 Feb 7;13:832403. doi: 10.3389/fphys.2022.832403 (PMC8859440; doi:10.3389/fphys.2022.832403)
Supplement: Supplementary file 1 [file Data_Sheet_1.PDF]

Supplementary Material

Detecting *de novo* Hepatic Ketogenesis Using Hyperpolarized [2-^{13}C] Pyruvate

Mukundan Ragavan[1,†], Marc A. McLeod[1,†], Anna Rushin[1], and Matthew E. Merritt[1*]

[1]Department of Biochemistry & Molecular Biology, College of Medicine, University of Florida, Gainesville, Florida, USA

[†]**equal contribution**

***Correspondence:**
Corresponding Author: matthewmerritt@ufl.edu

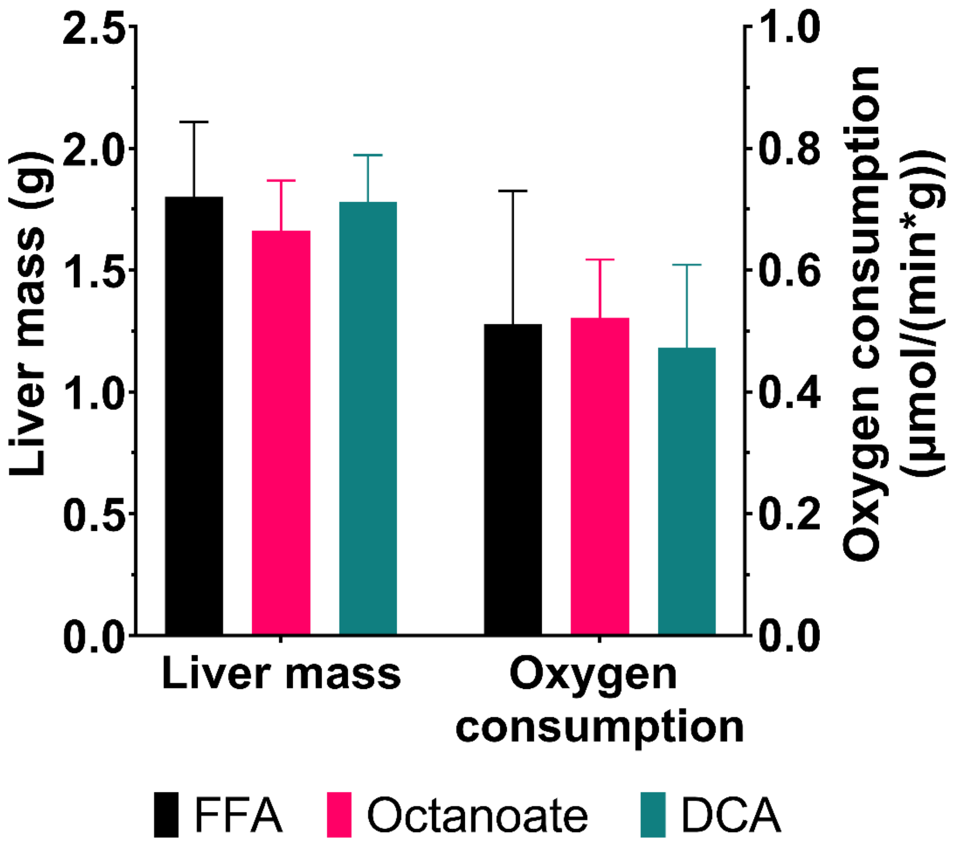

Figure S1: Liver mass (left) and hepatic oxygen consumption during perfusion (right) across all three groups. n = 5 (DCA, FFA) and 4 (octanoate). No significant differences are present between groups.

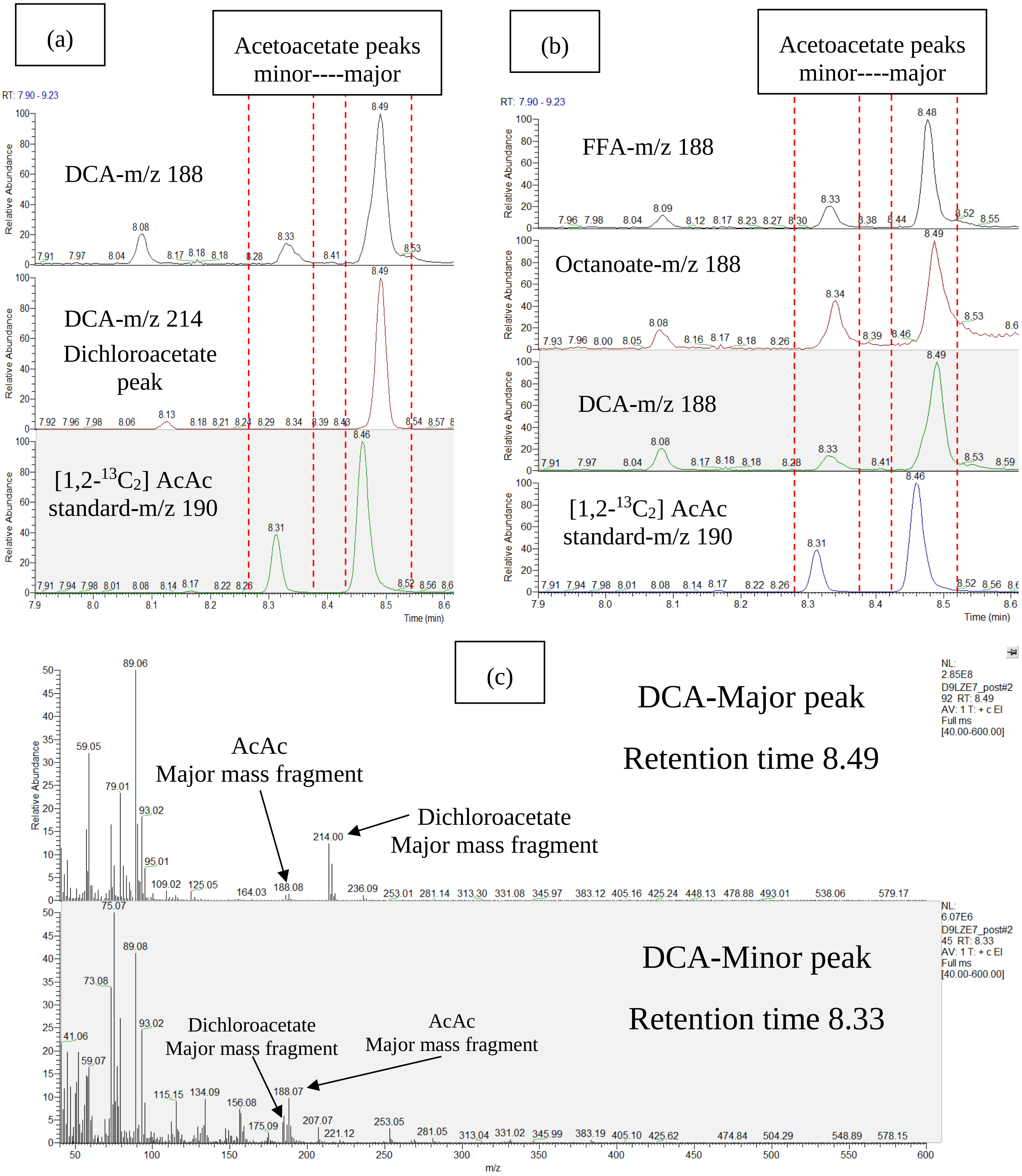
(a)
Acetoacetate peaks
minor----major
RT: 7.90 - 9.23
DCA-m/z 188
DCA-m/z 214
Dichloroacetate
peak
[1,2-$^{13}C_2$] AcAc
standard-m/z 190
Relative Abundance
Time (min)
(b)
Acetoacetate peaks
minor----major
RT: 7.90 - 9.23
FFA-m/z 188
Octanoate-m/z 188
DCA-m/z 188
[1,2-$^{13}C_2$] AcAc
standard-m/z 190
Relative Abundance
Time (min)
(c)
DCA-Major peak
Retention time 8.49
AcAc
Major mass fragment
Dichloroacetate
Major mass fragment
NL:
2.85E8
D9LZE7_post#2
92 RT: 8.49
AV: 1 T: + c EI
Full ms
[40.00-600.00]
DCA-Minor peak
Retention time 8.33
Dichloroacetate
Major mass fragment
AcAc
Major mass fragment
NL:
6.07E6
D9LZE7_post#2
45 RT: 8.33
AV: 1 T: + c EI
Full ms
[40.00-600.00]
Relative Abundance
m/z

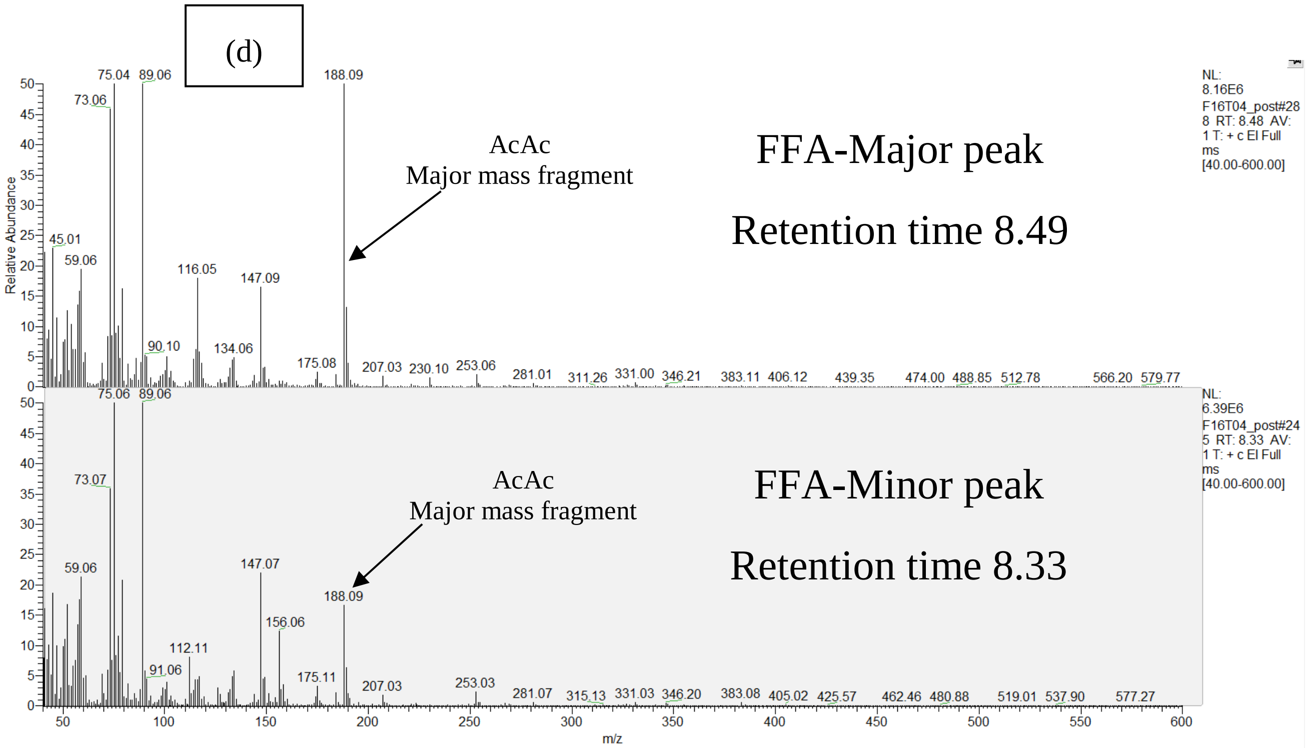

Figure S2:

(a) Stack of GC-MS extracted ion chromatogram for DCA perfusate m/z 188, m/z 214 and [1,2-$^{13}C_2$] acetoacetate standard-m/z 190 plotted in order. The red dashed lines indicate the minor (left) and major (right) acetoacetate peaks. Dichloroacetate has peaks corresponding to m/z 214 and m/z 186. Due to the distribution of chlorine isotopes (75% ^{35}Cl and 25% ^{37}Cl), DCA has spectral overlap at the major peak for AcAc at m/z 188.

(b) Stack of extracted ion chromatogram for FFA, Octanoate, DCA and [1,2-$^{13}C_2$] acetoacetate standard demonstrates the quantification of AcAc minor peak. DCA group has the smallest amount making it difficult to quantify isotopologues larger than M+1.

(c) Stack of mass spectra of major and minor AcAc peak regions from perfusates in DCA group. Dichloroacetate has a significant contribution to the major peak thereby confounding mass fragments preventing reliable integration of the AcAc peak at m/z 188. Minor peak, which does not have any contribution from dichloroacetate at m/z 188, can be reliably quantified.

(d) Stack of mass spectra for perfusates in FFA group showing major and minor AcAc peak regions at m/z 188 without any interference from other perfusate components (e.g. DCA as discussed in (c)).

Table S1: Metabolites derivatized by Methoxyamine hydrochloride and dimethyl tert butyl silyl trifluoroacetamide (MBSTFA) and their associated quantitation ions for GC-MS identification

**Metabolite**	**m/z Quantitation Ion**
Pyruvate	174-179
Lactate	261-267
Alanine	260-264
3-hydroxybutyrate	275-279
Acetoacetate	188-190
Succinate	289-293
Fumarate	287-290
Malate	419-423
Aspartate	418-422
Glutamate	432-436
Citrate	459-463
